# Anti‐desmoglein‐2 autoantibodies do not discriminate between UK boxer dogs with and without arrhythmogenic right ventricular cardiomyopathy

**DOI:** 10.1002/vetr.6014

**Published:** 2025-12-06

**Authors:** Chia‐Hsuan Chang, Claire Watson, Diptendu Chatterjee, Jade Ward, Kieran Borgeat, Hannah Hodgkiss‐Geere, Joanna Dukes‐McEwan, Robert Hamilton, Melanie J. Hezzell

**Affiliations:** ^1^ Bristol Veterinary School University of Bristol Langford UK; ^2^ Langford Vets University of Bristol Langford UK; ^3^ Labatt Family Heart Centre, The Hospital for Sick Children & Research Institute University of Toronto Toronto Ontario Canada; ^4^ Department of Small Animal Clinical Science, Institute of Infection, Veterinary and Ecological Sciences University of Liverpool Neston UK; ^5^ Present address: Rouken Glen Veterinary Surgery Glasgow UK; ^6^ Present address: Bristol Vet Specialists Bristol UK

**Keywords:** arrhythmias, biomarkers, cardiology, cardiomyopathies, cardiovascular diseases

## Abstract

**Background:**

Evidence regarding the diagnostic utility of serum anti‐desmoglein‐2 (DSG2) autoantibodies for arrhythmogenic right ventricular cardiomyopathy (ARVC) in boxer dogs is conflicting.

**Methods:**

Prospective standardised evaluation of apparently healthy boxer dogs for ARVC was performed at three referral centres, including blood pressure measurement, electrocardiography, echocardiography, haematology, biochemistry (including cardiac troponin I) and 24‐hour Holter monitoring. Additional dogs with a diagnosis of ARVC were retrospectively recruited. ARVC disease status was defined using cut‐offs of 20 or less (unaffected) and more than 300 (affected) ventricular premature complexes of right ventricular origin in 24 hours. The residual serum samples were stored at ‒80°C for analysis for anti‐DSG2 autoantibodies using ELISA techniques.

**Results:**

Forty boxer dogs were enrolled (11 healthy controls, 10 with preclinical ARVC and 19 with clinical ARVC). Serum anti‐DSG2 autoantibodies were detected in all dogs, bar one healthy dog. DSG2 differed significantly between groups (*p* = 0.031) and was significantly lower in dogs with preclinical versus clinical ARVC (*p* = 0.025).

**Limitations:**

Some data were collected retrospectively, and some dogs were receiving antiarrhythmic therapy.

**Conclusion:**

Serum DSG2 autoantibodies can be present in boxer dogs with preclinical and clinical ARVC and apparently healthy controls.

## INTRODUCTION

Arrhythmogenic right ventricular cardiomyopathy (ARVC) is a heritable cardiomyopathy in boxer dogs characterised by progressive fibrofatty replacement of the myocardium, primarily in the right ventricle, although the left ventricle may also be affected.^1^ This disease is associated with arrhythmias, which may lead to sudden cardiac death and/or ventricular dysfunction. The expression of the disease in dogs varies from preclinical disease (i.e., not associated with clinical signs, similar to Harpster category 1 boxers)^2^ to clinical disease, which can be subdivided into arrhythmias resulting in clinical signs such as syncope (Harpster category 2 dogs) and clinical signs due to myocardial dysfunction or a dilated cardiomyopathy phenotype (Harpster category 3, which may result in left‐ and/or right‐sided congestive heart failure).^3^

The antemortem diagnosis of ARVC is challenging, as histopathology is required for definitive confirmation. The clinical diagnosis of ARVC in dogs is currently based on family history and clinical presentations supported by a set of clinical tests including electrocardiography (ECG), continuous Holter ECG monitoring and echocardiography.^1^, ^4^ Holter monitoring is valuable for the identification of preclinical cases of ARVC, in particular assessing the degree of ‘silent’ ventricular ectopy. Nevertheless, the usefulness of this test is limited by its cost and an absence of a clear cut‐off to distinguish healthy dogs from affected dogs. Although cardiac troponin I is increased in boxer ARVC, the sensitivity for disease detection is suboptimal, with overlap between affected and unaffected dogs.^5^ The development of a straightforward, accurate and low‐cost diagnostic test would therefore have clear clinical value for the identification of early ARVC in dogs.

Desmoglein‐2 (DSG2) is a desmosomal protein that is localised to the intercalated discs in cardiac myocytes; therefore, it is important in maintaining cell‐to‐cell adhesion. Mutations in DSG2 are associated with ARVC and familial dilated cardiomyopathy in human patients. One study suggested that measurements of serum autoantibodies against DSG2 are a highly sensitive and specific biomarker in human patients and boxer dogs with clinical ARVC.^6^ However, a subsequent study conducted in the United States demonstrated that anti‐DSG2 autoantibodies could be measured in healthy boxers and measurements did not differ between boxers with ARVC, healthy control dogs (boxers and non‐boxers), Doberman pinschers with dilated cardiomyopathy and small breed dogs with myxomatous mitral valve disease.^7^, ^8^ The present study aimed to further investigate this discrepancy in the literature using a UK population of boxers. The study objectives were to quantify serum anti‐DSG2 autoantibodies in boxer dogs and to compare measurements between healthy control, preclinical ARVC and clinical ARVC groups. The study hypothesis was that measurements of serum anti‐DSG2 autoantibodies would differ between healthy boxer dogs, boxer dogs with preclinical ARVC and those with clinical ARVC.

## MATERIALS AND METHODS

### Study design

The study employed an observational design and convenience sampling; both prospective and retrospective recruitments were used.

The study inclusion criteria were that all dogs must be pedigree boxer dogs (based on a Kennel Club registration certificate) with no evidence of significant systemic disease (e.g., neoplastic, immune‐mediated or infectious disease), based on physical examination, blood pressure measurement and the results of complete blood counts and serum biochemistry.

Systolic blood pressure was measured by Doppler ultrasound with a sphygmomanometer (Parks model 811 B, Parks Medical Electronics Inc.) and the size of the pressure cuff closest to 40% of the circumference of the dog's antebrachium was placed at the mid‐radius.

ARVC was diagnosed or excluded by a board‐certified cardiologist based on a combination of clinical signs, ECG findings, echocardiography and 24‐hour Holter ECG results. A 3‐minute, 12‐lead ECG (EasyECG Pocket, EBNeuro, or Cardiofax V, Nihon Kohden) was recorded with each dog gently restrained in right lateral recumbency and the paper speed was set at 50 mm/s. Heart rate, rhythm and other measurements were assessed from lead II and chest leads.

Echocardiographic examinations were performed by a specialist in veterinary cardiology using an ultrasound unit (Vivid iQ, GE Healthcare, or Vivid E95, GE Healthcare) equipped with 3‒4 MHz matrix transducer and ECG monitoring. Each dog was gently restrained in right then left lateral recumbency on an examination table. All standardised imaging planes and images were optimised and digitally stored. Assessment of overall cardiac morphology and size was performed from the right parasternal long‐axis view. Measurements of systolic and diastolic left ventricular (LV) dimensions and short‐axis diastolic LA:Ao ratio in early diastole were recorded. LV fractional shortening was calculated using the formula: [(LV end‐diastolic diameter ‒ LV end‐systolic diameter)/LV end‐diastolic diameter] × 100. The left heart measurements were recorded and compared to published reference values.^9^, ^10^ The left apical four‐chamber view was obtained allowing measurements of trans‐mitral velocities. Valvular insufficiency was assessed by colour Doppler blood flow across each valve, and was classified as mild, moderate and severe if valvular disease was present. Dogs with moderate or severe valvular regurgitation were excluded.

In dogs for which data were collected prospectively, the Holter monitor unit (Trillium 7000, Forest Medical) was attached to each dog and was set to record for 48 hours. The owner was asked to keep a diary of each dog's activities throughout the recording period. The recorded ECG was reviewed and interpreted by a single investigator (K.B.) blinded to all clinical findings using commercially available Holter analysis software (Trillium Platinum Vet, Forest Medical). For data collected retrospectively, Holter analysis performed using a different system was included (Holter monitor unit; Lifecard CF, Holter analysis software; Spacelabs Pathfinder, both Spacelabs Healthcare).

All examinations were performed without sedation. Boxer dogs that met the following criteria were categorised in the healthy control group: no history of ARVC‐related clinical signs, no pathological murmur, no evidence of structural heart disease on echocardiography, no electrical abnormalities on a 3‐minute surface ECG recording, 20 or less ventricular premature complexes (VPCs) on a 24‐hour Holter ECG recording and no evidence of clinically significant systemic disease on blood tests.^4^ Boxers were diagnosed with ARVC if more than 300 VPCs of right ventricular origin/24 hours were recorded during a Holter ECG recording.^4^ Dogs were not excluded if there was some variation in the morphology of the VPCs during the Holter recording (e.g., related to changes in the dog's position). The severity of arrhythmia was assessed using a previously reported scale,^11^, ^12^ as follows: 0 = no VPC, 1 = single VPC, 2 = bigeminy or trigeminy, 3 = couplets or triplets and 4 = R‐on‐T phenomenon or ventricular tachycardia. Dogs with ARVC were subdivided according to disease severity into preclinical and clinical groups. Preclinical cases were defined as dogs without clinical signs referable to their cardiac disease. Clinical cases were defined as dogs in which clinical signs consistent with ARVC were reported (e.g., syncope). Dogs with clinical ARVC that were receiving cardiac medications were included in this study. Exclusion criteria included any clinically significant systemic disease with an influence on the cardiovascular system or on volume homeostasis, treatment with any drugs with influence on volume homeostasis (e.g., corticosteroids) and evidence of any cardiovascular disease other than ARVC.

Apparently healthy, client‐owned boxer dogs and boxer dogs previously diagnosed with ARVC were prospectively recruited by Langford Vets (University of Bristol), the University of Liverpool and the Ralph Veterinary Referral Centre and underwent diagnostic testing as described above (prospective group). Blood was collected from a vein, divided between serum and K‐EDTA containers and haematology, serum biochemistry and cardiac troponin I levels were measured by the Langford Vets Diagnostic Laboratory. Residual serum samples were stored at ‒80°C until the time of analysis.

Additional boxer dogs that had previously been diagnosed with ARVC at Langford Vets were identified from electronic patient records (retrospective group). The Langford Vets archive of residual serum samples was searched to identify boxer dogs for which residual serum samples, collected contemporaneously with diagnostic testing for ARVC, were available. In some of the retrospectively identified dogs, this serum sample was not collected at the time of initial diagnosis; dogs that were receiving antiarrhythmic therapy at the time of serum collection were not excluded from the study.

Owners gave informed written consent for samples to be used for research in all cases. The samples were transported on dry ice to the University of Toronto for batched analysis of serum anti‐DSG2 autoantibodies using an ELISA technique.

### DSG‐2 antibody ELISA

A direct ELISA was performed as previously described.^6^, ^8^ Briefly, a microtitre plate (Corning Costar 96‐Well EIA Plate #2592; ICT #25, Immunochemistry Technologies) was first coated with 100 µL (at 2 µg/mL dilution in antigen coating buffer 5×: Cat no. 6247, Immunochemistry Technologies) of recombinant human DSG2 protein (Creative Biomart, Cat no. DSG2‐512H). The wells were blocked with 5% bovine serum albumin in Tris‐buffered saline containing 0.1% Tween 20 (TBST) and then exposing each well to diluted human sera (100 µL of 1:100 dilutions in Neptune sample diluent, Cat no. 6124, Immunochemistry Technologies) followed by washing three times with TBST. The resultant bound anti‐DSG2 antibody was then assayed by anti‐dog IgG‐horseradish peroxidase (at 1:20,000 dilutions, Abcam, Cat no. ab112840) using tetramethylbenzidine chromophore to measure optical density (O.D.) at 450 nm wavelength. The ELISA test result was considered negative if the O.D. value from each sample was less than 0.75. Validation of the ELISA was performed using both monoclonal and commercially available polyclonal antibodies against DSG2 (Rabbit Polyclonal Desmoglein 2/DSG2 antibody and Anti‐Desmoglein 2/DSG2 antibody [Abcam]). Results of spiking recovery demonstrated good assay accuracy.

### Statistical analysis

All the data were recorded and collated into an electronic database (Excel) and statistical analysis was performed using commercially available software (SPSS 24, IBM United Kingdom Limited and GraphPad Prism v10.3.0, GraphPad Software). The normality of data was assessed by visual inspection (histograms or Q‒Q plots) and the use of Shapiro‒Wilk tests. Summary data are reported as mean ± standard deviation for normally distributed data and median (minimum, maximum) for data that were non‐normally distributed. Categorical variables were reported as proportions. One‐way ANOVA tests (for normally distributed data) and Kruskal‒Wallis tests (for non‐normally distributed data) were used for comparisons of continuous variables across groups, while categorical variables were compared using chi‐square test or Fisher's exact tests, as appropriate. Pairwise post hoc comparisons were performed between groups using Dunn's tests for multiple comparisons (reported as adjusted *p*‐values). Dogs for which Holter recording were not available for review were excluded from analyses of electrocardiographic changes but included in all other analyses. Simple linear regression analysis was performed to evaluate the relationship between the frequency of VPCs and serum anti‐DSG2 autoantibody O.D. To minimise the influence of antiarrhythmic medications on frequency of arrhythmias, dogs receiving antiarrhythmic medications were excluded from the linear regression analysis. A sample size calculation performed using commercially available software (Power and Sample Size calculator, Vanderbilt University) suggested that 20 affected boxers and 20 control dogs are required to demonstrate a difference of 55% in the proportion of dogs with detectable DSG2 autoantibodies between groups with a power of 80% and an alpha of 0.05. *p*‐Values of less than 0.05 were considered significant.

## RESULTS

Forty boxer dogs were enrolled in the study. Fifteen dogs were retrospectively identified from the electronic patient record, and 25 dogs were prospectively recruited between March and November 2021, including 11 healthy control dogs (nine recruited prospectively), 10 dogs with preclinical ARVC (nine recruited prospectively) and 19 dogs with clinical ARVC (seven recruited prospectively). All dogs in the clinical group had Harpster category 2 disease (clinical signs associated with arrhythmias and no evidence of systolic dysfunction or congestive heart failure). Retrospectively recruited dogs were assessed by one of five board‐certified cardiologists, or supervised residents in training at Langford Vets. Twenty prospectively recruited dogs were assessed by a single board‐certified cardiologist (M.J.H.) at Langford Vets, four dogs were assessed by one of two board‐certified cardiologists at the University of Liverpool, and one dog was assessed by a cardiologist at the Ralph Veterinary Referral Centre.

Descriptive statistics summarising the age, weight, sex distribution, clinical presentation, heart rate, blood pressure and grade of heart murmur for each group are presented in Table 1.

**TABLE 1:** Comparison of characteristics and clinical data among healthy dogs and dogs with different stages of arrhythmogenic right ventricular cardiomyopathy (ARVC).

Variable	Healthy control	Preclinical ARVC	Clinical ARVC	*p*‐Value
Age (years)	7.81 ± 1.90 (*n* = 11)	8.35 ± 1.66 (*n* = 10)	8.13 ± 2.07 (*n* = 19)	0.810
Weight (kg)	30.34 ± 3.79 (*n* = 11)	29.55 ± 4.81 (*n* = 10)	31.69 ± 4.85 (*n* = 19)	0.465
Sex				
Male (neutered)	6 (2)	5 (1)	12 (5)	0.772 (0.801)
Female (spayed)	5 (4) (*n* = 11)	5 (3) (*n* = 10)	7 (5) (*n* = 19)	
Heart rate (beats per minute)	123.60 ± 16.13 (*n* = 10)	127.40 ± 32.55 (*n* = 10)	130.94 ± 44.60 (*n* = 18)	0.874
Systolic blood pressure (mmHg)	168.33 ± 24.45^a^	180.13 ± 17.72^b^	139.36 ± 27.40^a,b^	**0.02**
Heart murmur grade	0 (0, 3)	2 (0, 3)	1 (0, 4)	0.569

*Note*: *p*‐Values for variables for which significant between‐group differences were detected (*p* < 0.05) are highlighted in bold text. Matching pairs of superscript letters denote significant differences in post hoc groupwise comparisons.

Age, weight, heart rate and blood pressure were normally distributed. The grade of heart murmur was non‐normally distributed. No differences were detected in age and bodyweight across groups (*p* = 0.810 and 0.465, respectively). Data from 23 of 40 (57.5%; 95% confidence interval [CI]: 42.20‒72.8) male dogs and 17 of 40 (42.5%; 95% CI: 27.2‒57.8) female dogs were included. No significant differences were detected in the proportions of sex and neuter status across groups (*p* = 0.772 and 0.801, respectively). No significant difference in heart rate was detected across study groups (*p* = 0.874). A significant difference was found in systolic blood pressure across groups (*p* = 0.020). Systolic blood pressure was significantly lower in the clinical ARVC group compared to both the healthy and preclinical ARVC groups (adjusted *p* = 0.026 and 0.002, respectively). Heart murmurs were present in 23 of 40 (57.5%; 95% CI: 42.2‒72.8) of the total population. All murmurs were echocardiographically confirmed to be physiological ejection murmurs; no dog had evidence of structural heart disease (e.g., subaortic stenosis). There was no difference in heart murmur grade across all groups (*p* = 0.569). Syncope was the most commonly reported clinical sign in dogs with ARVC in 16 of 29 (55.2%; 95% CI: 39.8‒70.6) dogs, followed by exercise intolerance in 15 of 29 (51.7%; 95% CI: 36.2‒67.2) dogs.

Dogs receiving medications were included in the study. In the control group, one dog received paracetamol for pain management (healed limb fractures). In the clinical group, every dog received antiarrhythmic medication (14/19 [73.7%; 95% CI: 0.539‒0.935] received sotalol alone, two received sotalol and mexiletine, one received mexiletine alone and two received amiodarone and diltiazem), seven dogs received pimobendan, four dogs received angiotensin‐converting enzyme inhibitors and four dogs received furosemide. Nine dogs received more than one medication. The median dosage of sotalol was 3.15 mg/kg/day (minimum = 2.26, maximum = 6.96).

Three‐minute ECG and 24‐hour Holter monitoring were performed on 40 dogs. However, the Holter monitoring of two dogs (one dog from the healthy group and one dog from the clinical group) was performed using the Spacelabs system and the raw data were not available for review. Information from the Spacelabs Holter reports was used to assign dogs to groups, but these dogs were excluded from statistical analysis of Holter findings due to missing data. The Holter data are presented in Table 2.

**TABLE 2:** Descriptive statistics summarising electrocardiography (ECG) rhythm, abnormalities and frequency and severity of ventricular arrhythmias during 3‐minute ECG and 24‐hour Holter monitoring across healthy boxer dogs and boxer dogs affected with different stages of arrhythmogenic right ventricular cardiomyopathy (ARVC).

Group	Healthy control (*N* = 10)	Preclinical ARVC (*N* = 10)	Clinical ARVC (*N* = 18)	*p*‐Value
3‐Minute ECG				
Rhythm				
Sinus rhythm	4	7	9	0.508
Sinus arrhythmia	5	2	7	
Sinus tachycardia	1	0	2	
Presence of VPCs	0	7	17	**<0.001**
24‐Hour Holter monitoring				
VPC counts per day	9.0 (0.0, 20.0)	3209.2 (366.0, 34,058.0)	1016.0 (2.0, 55,339.0)	**<0.001**
VA severity grading	1.0 (0.0, 3.0)	3.5 (3.0, 4.0)	3.0 (1.0, 4.0)	**<0.001**
SVE counts per day	0.0 (0.0, 1.0)	0.0 (0.0, 30.0)	530.0 (0.0, 36,012.0)	**<0.001**
Presence of SVT	0	0	5	N/A

*Note*: All variables included 10 dogs from the healthy group, 10 dogs from the preclinical and 18 dogs from the clinical group, except the following variables, which included nine healthy dogs, eight preclinical dogs and seven clinical dogs: SVE counts per day and episodes of SVT. Data for continuous variables are summarised as median (minimum, maximum) values. The *p*‐values for variables for which significant between‐group differences were detected (*p* < 0.05) are highlighted in bold text.

Abbreviations: N/A, not available; SVE, supraventricular ectopic complex; SVT, supraventricular tachyarrhythmia; VA, ventricular arrhythmia; VPC, ventricular premature complex.

No significant difference was detected between underlying heart rhythms on 3‐minute ECG across groups. VPCs were detected on 3‐minute ECG in 17 of 18 (94.4%; 95% CI: 87.3‒100) of the dogs in the clinical group. A significant difference in the proportion of dogs with VPCs recorded during the 3‐minute ECG was detected across groups (*p* < 0.001). On 24‐hour Holter monitoring, significant differences were detected in VPC counts per day, ventricular arrhythmia severity and supraventricular ectopic complexes per day (all adjusted *p* < 0.001). As expected, the VPC counts per day of the healthy dogs were significantly lower than dogs in the preclinical and clinical groups (both adjusted *p* < 0.001); no significant difference in VPC counts per day was detected between dogs in the preclinical and clinical groups (adjusted *p* = 0.642). The ventricular arrhythmia severity score was also lower in the healthy dogs compared with the dogs from the preclinical and clinical groups (both adjusted *p* < 0.001); no significant difference in ventricular arrhythmia score was detected between dogs in the preclinical and clinical groups (adjusted *p* > 0.999). The number of supraventricular ectopic complexes was significantly higher in the clinical group than the healthy (adjusted *p* = 0.007) and preclinical (adjusted *p* = 0.006) groups; no significant difference in the number of supraventricular ectopic complexes was detected between dogs in the healthy and preclinical groups (adjusted *p* > 0.999).

**FIGURE 1:**
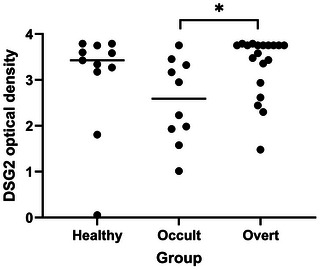
Comparison of serum anti‐desmoglein‐2 autoantibody optical density value across healthy boxer dogs, and boxer dogs affected with different stages of arrhythmogenic right ventricular cardiomyopathy (ARVC). An overall difference among groups was detected (*p* = 0.031). Post hoc pairwise comparisons with Dunn's test demonstrated a significant difference between the preclinical and the clinical groups (*p* = 0.025). No significant differences were detected between any other pair of groups. ^*^ *p* = 0.025.

The serum anti‐DSG2 autoantibody O.D. was measured in 40 dogs (Figure 1). There was a significant difference in anti‐DSG2 autoantibody O.D. among groups (*p* = 0.031). The serum anti‐DSG2 autoantibody O.D. was significantly higher in the clinical group than the preclinical group (adjusted *p* = 0.019). No differences were detected between any other pair of groups. A negative result was only recorded from one dog, which was in the healthy control group. Twenty dogs (11 healthy and nine preclinical dogs) were included in the model (dependent variable: serum anti‐DSG2 autoantibody O.D.). No significant relationship was found between the frequency of VPCs and serum anti‐DSG2 autoantibody O.D. (*p* = 0.081, *B* = ‒2806.3 [95% CI for *B* = ‒5986.0 to 373.4]).

## DISCUSSION

The main aim of the current study was to evaluate the utility of serum anti‐DSG2 autoantibody detection for the diagnosis of both preclinical and clinical ARVC in a UK population of boxer dogs; the results are not, therefore, generalisable to boxer dogs outside the UK or other breeds of dog. Although anti‐DSG2 autoantibody measurement (using Western blot techniques) has previously been reported to be highly sensitive and specific in boxers with clinical ARVC, more recent studies conducted in the United States, have demonstrated that serum anti‐DSG2 autoantibody measurements do not differentiate healthy dogs from affected boxer dogs^7^, ^8^ and that anti‐DSG2 autoantibodies are detectable in Doberman pinschers with dilated cardiomyopathy and dogs with myxomatous mitral valve disease.^7^ In the present study, the serum anti‐DSG2 O.D. value was significantly higher in dogs with clinical ARVC than those with preclinical ARVC. However, the serum anti‐DSG2 autoantibody O.D. were similar between the healthy group and the clinical group and serum anti‐DSG2 autoantibodies were detected in every dog in the healthy group bar one, in agreement with an earlier study.^7^ Measurement of serum anti‐DSG2 autoantibodies was not therefore clinically useful, as it did not differentiate the healthy from the preclinical group and the clinical group can be readily distinguished from the preclinical group based on clinical presentation.

No significant relationship was found between the O.D. of serum anti‐DSG2 autoantibodies and the frequency of VPCs (*p* = 0.081). This differs from findings previously reported in human and boxer ARVC, in which serum anti‐DSG2 autoantibody measurements were positively correlated with VPC counts,^6^, ^8^ but this might reflect the small sample size, especially following exclusion of dogs receiving antiarrhythmic medication. It is interesting to note that the VPC count in dogs with other cardiac diseases correlated with anti‐DSG2 concentration in a previous study.^8^ It is possible that anti‐DSG2 reflect sequelae of myocardial and electrical remodelling in a variety of diseases as recently proposed.^13^

Several modifications could be made to the canine serum anti‐DSG2 autoantibody test prior to re‐evaluation. Specifically, to ensure that the test is clinically translatable, the human anti‐DSG2 test has been redeveloped using only peptides from three extracellular cadherin domains in the ELISA. This avoids the use of complex blocking steps to avoid reaction with the Fc tag that was needed to solubilise the whole extracellular DSG2 protein, as the shorter peptides are intrinsically soluble. It is likely that a canine test could be developed by using the sequences of the canine DSG2 extracellular cadherin domains.

Male dogs affected with ARVC were overrepresented in most previous studies,^3^, ^14^ although a higher proportion of female dogs have also been reported.^11^ There was no sex predisposition in this study, and no significant difference in the mean ages of healthy controls and ARVC dogs was detected. This was expected since the study inclusion criteria included age. Syncope, followed by exercise intolerance, was the most commonly reported clinical sign, which is similar to previous reports of ARVC in boxer dogs.^11^ Heart rate was not significantly different across groups. Systolic blood pressure was found to be higher in the healthy and the preclinical dogs than in the clinical group. It is possible that dogs in the clinical group were developing systolic dysfunction, despite echocardiographic measurements being within normal reference intervals (data not shown); however, measurements could be influenced by several factors, including stress, excitement and medications (e.g., the beta blocking effects of sotalol) in the present study. The presence and grade of heart murmur were also not different across groups in the present study; this is probably due to the frequency with which non‐pathological left basilar ejection murmurs are detected in the boxer breed.^15^

In this relatively small sample of boxers, the presence of VPCs on the 3‐minute ECG recording was only seen in dogs diagnosed with ARVC based on 24‐hour Holter ECG analysis. The diagnostic utility of a 3 or 5‐minute ECG in screening boxers for ARVC could be investigated in a larger population of dogs.

There are several limitations to this study. First, the results are based on a combination of prospectively and retrospectively collected case material; datasets were incomplete from most dogs recruited retrospectively. Due to the limitations of historical data collection, complete electrocardiographic and echocardiographic assessments were not available in every dog. Additionally, all dogs with clinical disease were already receiving treatment at the time of assessment for the study. Another limitation is that it is impossible to definitively diagnose ARVC antemortem, as histopathology is required. However, the clinical diagnosis of ARVC is based on clinical signs, ECG or Holter results, and echocardiographic abnormalities. In this study, the diagnosis of ARVC was mainly based on the frequency and complexity of ventricular arrhythmias, which could be affected by the significant day‐to‐day variability observed in boxer dogs.^16^ To minimise the risk of misclassification, we chose to include dogs above the average age of onset of clinical signs or dogs related to affected dogs. Additionally, the definitions of healthy and affected dogs, which are based on the number of VPCs/24 hours on a Holter ECG recording were relatively stringent, with dogs with counts between 21 and 300 VPCs/24 hours being excluded from the study.

## CONCLUSIONS

The study demonstrated that serum anti‐DSG2 autoantibody O.D. was lower in boxer dogs with preclinical compared to those with clinical ARVC. However, anti‐DSG2 autoantibodies were also detected in the serum of healthy boxers. This suggests that serum anti‐DSG2 autoantibody measurement is not a suitable diagnostic tool for the identification of ARVC in boxer dogs.

## AUTHOR CONTRIBUTIONS

Chia‐Hsuan Chang made substantial contributions to data acquisition, analysis and interpretation and drafted the manuscript. Jade Ward, Hannah Hodgkiss‐Geere and Joanna Dukes‐McEwen made substantial contributions to data acquisition and critically revised the manuscript. Kieran Borgeat and Robert Hamilton made substantial contributions to the study conception and design and data acquisition and critically revised the manuscript. Claire Watson made substantial contributions to the study conception and design and critically revised the manuscript. Diptendu Chatterjee made substantial contributions to the acquisition of data, analysis and interpretation of data and critically revised the manuscript. Melanie Hezzell made substantial contributions to the study conception and design and data acquisition, analysis and interpretation and critically revised the manuscript. All the authors have given final approval for the version to be published. Each author has participated sufficiently in the work to take public responsibility for appropriate portions of the content and agrees to be accountable for all aspects of the work.

## CONFLICT OF INTEREST STATEMENT

The authors declare no conflicts of interest.

## ETHICS STATEMENT

The study protocol was reviewed and approved by the University of Bristol Animal Welfare and Ethical Review Body (VIN/18/027), and informed written owner consent was obtained for all dogs.

## Data Availability

The data that support the findings of this study are available on request from the corresponding author. The data are not publicly available due to privacy or ethical restrictions.
